# Time Course of Priming Effect of TF Inducers on Synergistic TF Expression and Intra-Cellular Gap Formation of Human Vascular Endothelial Cells via the Extrinsic Coagulation Cascade

**DOI:** 10.3390/ijms241512388

**Published:** 2023-08-03

**Authors:** Daiki Matsubara, Takuma Kunieda, Yuhki Yanase, Shunsuke Takahagi, Kazue Uchida, Tomoko Kawaguchi, Kaori Ishii, Akio Tanaka, Koichiro Ozawa, Michihiro Hide

**Affiliations:** 1 Department of Dermatology, Graduate School of Biomedical and Health Sciences, Hiroshima University, Hiroshima 734-8551, Japan; daiki_matsubara_32_5@yahoo.co.jp (D.M.); takshuns@gmail.com (S.T.); uchidak@hiroshima-u.ac.jp (K.U.); tomokok@hiroshima-u.ac.jp (T.K.); ishiik@hiroshima-u.ac.jp (K.I.); tantanakiotan@yahoo.co.jp (A.T.); 2 Department of Pharmacotherapy, Graduate School of Biomedical and Health Sciences, Hiroshima University, Hiroshima 734-8551, Japan; m225029@hiroshima-u.ac.jp (T.K.); ozawak@hiroshima-u.ac.jp (K.O.); 3 Hiroshima City Hiroshima Citizens Hospital, Hiroshima 730-8518, Japan

**Keywords:** chronic spontaneous urticaria (CSU), tissue factor (TF), vascular endothelial cells, intra-cellular gap formation, extrinsic coagulation pathway, TF inducer (TFI)

## Abstract

Chronic spontaneous urticaria (CSU) is characterized by daily recurring wheal and flare with itch for more than 6 weeks. The extrinsic coagulation system has been shown to be activated in correlation with CSU severity. We have reported that tissue factor (TF), a trigger of the extrinsic coagulation cascade, is synergistically expressed on vascular endothelial cells by simultaneous stimulation with TF inducers (TFI), followed by activation of the extrinsic coagulation cascade and hyper permeability in vitro. However, vascular endothelial cells are not likely to be simultaneously stimulated by multiple TFIs under physiological conditions. Therefore, in order to know whether sequential, rather than simultaneous, stimuli with interval may induce synergistic activation of TF, we investigated the time course of the priming effects of each TFI for synergistic TF expression in vascular endothelial cells (HUVECs). We stimulated HUVECs with a TFI (first stimulation) and then stimulated cells with another TFI at indicated time points (second stimulation) and detected TF expression and activity. The TF expression induced by simultaneous stimulation diminished in a few hours. However, both synergistic enhancement of TF expression and activation level of the coagulation cascade were detected even when the second stimulation was added 18 or 22 h after the first stimulation. Thus, the priming effect of TFI for synergistic TF expression may persist for a half day or longer.

## 1. Introduction

Chronic spontaneous urticaria (CSU), which is also known as chronic idiopathic urticaria (CIU), is a common skin disease characterized by daily and/or almost daily recurring wheal and flare with itch induced by several inflammatory mediators, such as histamine, proteases lipid mediators, and proinflammatory cytokines, which are released from human mast cells in the skin and/or basophils in peripheral blood [1,2,3,4]. The increase in plasma histamine level in peripheral blood and the significant effect of H1 antihistamines imply an important role of histamine and their receptor, histamine H1 receptor (H_1_R), in the pathogenesis of CSU [5]. The presence of autoantibodies (IgG) against the IgE antibody and/or the high-affinity IgE receptor (FcεRI) expressed on mast cells in the skin and basophils in peripheral blood may be observed in around 40% of patients with CSU, suggesting the involvement of autoantibodies in the pathogenesis of CSU [6,7]. IgE antibodies against self molecules, such as double-stranded deoxyribonucleic acid (dsDNA), tissue factor (TF), thyroid peroxidase (TPO), and interleukin-24 (IL-24) have also been detected in sera of CSU patients [8,9,10]. Moreover, the rapid effect of monoclonal antibodies (IgG) against human IgE antibodies, such as omalizumab and ligelizumab, suggests the crucial roles of IgE antibodies against autoantigens [7,11]. However, the constitutional presence of autoantibodies and/or autoantigens does not explain diurnal time course and local occurrence of wheals recognized in CSU patients. Recently, the blood coagulation system and infection by bacteria and/or virus have also been considered to be associated with the pathogenesis of CSU [4,12,13,14]. Several reports revealed that heparin has inhibitory activity against blood coagulation factors, and may be effective in the treatment of CSU [15]. Moreover, warfarin, which suppresses the production of coagulation factors such as factor VII, factor IX, factor X, and factor II, may reduce symptoms of CSU that were unresponsive to antihistamines [16]. Several groups demonstrated that the levels of prothrombin fragment 1 + 2 (PF_1+2_) and D-dimer in plasma of CSU patients are higher than those in healthy controls and correlate with disease severities [17,18]. The elevation of activation potential of the extrinsic coagulation cascade in patients with CSU as compared to that in healthy donors was also reported [19]. However, a detailed mechanism of how the extrinsic coagulation cascade and infection affect dynamics of the pathogenesis of CSU has not been clear. We reported that histamine and lipopolysaccharide (LPS), an endotoxin in the outer membrane of Gram-negative bacteria, including *Helocobactor pylori*, synergistically increase expression level of TF mRNA and protein in human umbilical vein endothelial cells (HUVECs) in vitro [20]. Moreover, we clarified that a large amount of TF, expressed on the surface of HUVECs in response to simultaneous stimulation of histamine and LPS, activates the extrinsic coagulation cascade and produces active forms of coagulation factors, such as factor Xa and factor IIa, resulting in the induction of intercellular gap formation of HUVECs via protease activated receptor 1 (PAR-1) expressed on HUVECs [20]. We also clarified that the expression of TF mRNA and TF protein on the surface of HUVECs in response to histamine or vascular endothelial growth factor (VEGF), which activate the protein kinase C (PKC) pathway (Group 2 TF inducer (TFI)), were synergistically enhanced by treatment with tumor necrosis factor α (TNF-α), LPS, IL-1β, or IL-33, which activate the NF-κB dependent pathway (Group 1 TFI), whereas only additive effects were observed by costimulation with TFIs of the same group [21]. Furthermore, the intercellular gap of HUVECs induced by histamine or VEGF (Group 1 TFI) were also synergistically enhanced in the presence of TNF-α or LPS (Group 2 TFI) [21]. However, human vascular endothelial cells were not likely simultaneously stimulated with both groups of TFIs in physiological condition. Therefore, in order to know whether sequential, rather than simultaneous, stimuli with an interval may induce synergistic activation of TF, we examined the time course of the priming effects of TFIs, including LPS, TNF-α, VEGF, and histamine on the synergistic expression of TF in HUVECs and its activity as a trigger of the extrinsic coagulation cascade, production of active forms of coagulation factors, and intercellular gap formation of HUVECs in the presence of factor VIII-deficient human plasma containing extrinsic coagulation factors, such as factor Vll, factor X and factor II.

## 2. Results

### 2.1. Time-Dependent Expression Levels of TF mRNA in HUVECs by Single and Dual Stimulations with TFI

**Figure 1:**
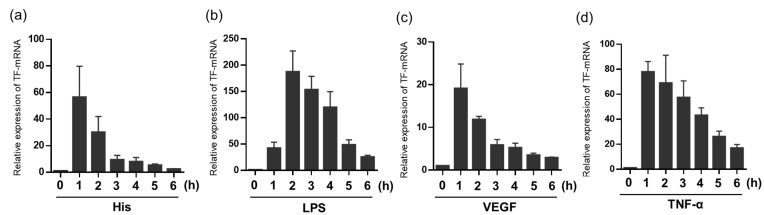
Time course of TF mRNA expression by stimulation with TFIs, histamine, VEGF, LPS, and TNF-α in HUVECs. Relative expression levels of TF mRNA in response to each TFI, (**a**) Histamine, (**b**) LPS, (**c**) VEGF, (**d**) TNF-α at indicated time points. Concentrations of each molecule are VEGF (10 ng/mL), TNF-α (10 ng/mL), LPS (100 ng/mL), and/or histamine (10 μM). All figures show results of four or five independent three experiments. Data represent mean ± standard error.

We first confirmed the time course of TF mRNA expression stimulated with each TFI, LPS, TNF-α, histamine, or VEGF in HUVECs. As shown in Figure 1, TF mRNA expression levels in HUVECs in response to each stimulus were quickly increased within 1 or 2 h and then gradually decreased to base line within a few hours (Figure 1).

**Figure 2:**
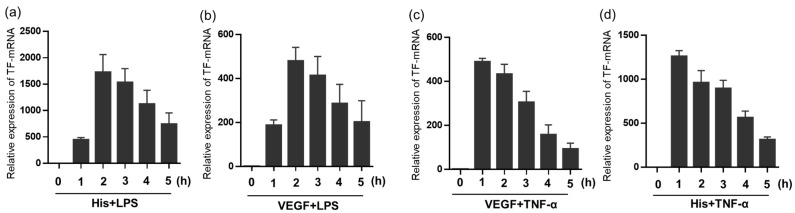
Time course of TF mRNA expression by stimulation with the combination of TFIs in HUVECs. Relative expression levels of TF mRNA at indicated time points in response to simultaneous stimulation of TFIs, (**a**) Histamine + LPS, (**b**) VEGF + LPS, (**c**) VEGF + TNF-α, (**d**) Histamine + TNF-α. Concentrations of each molecule are VEGF (10 ng/mL), TNF-α (10 ng/mL), LPS (100 ng/mL) and/or histamine (10 μM). All figures show results of three or four independent experiments. Data represent mean ± standard error.

We then investigated the time course of TF mRNA expression by simultaneous stimulation of Group 1 and Group 2 TFIs. We previously defined synergistic expression of TF mRNA induced by two TFIs as the synergistic expression/additive expression (S/A) ratio is greater than two [21] (Section 4). As shown in Figure 2, the synergistic expression of TF mRNA was also observed within 1 or 2 h, using TNF-α or LPS as a reagent in Group 1 TFI with either histamine or VEGF as a reagent in Group 2 TFI, and then gradually decreased.

**Figure 3:**
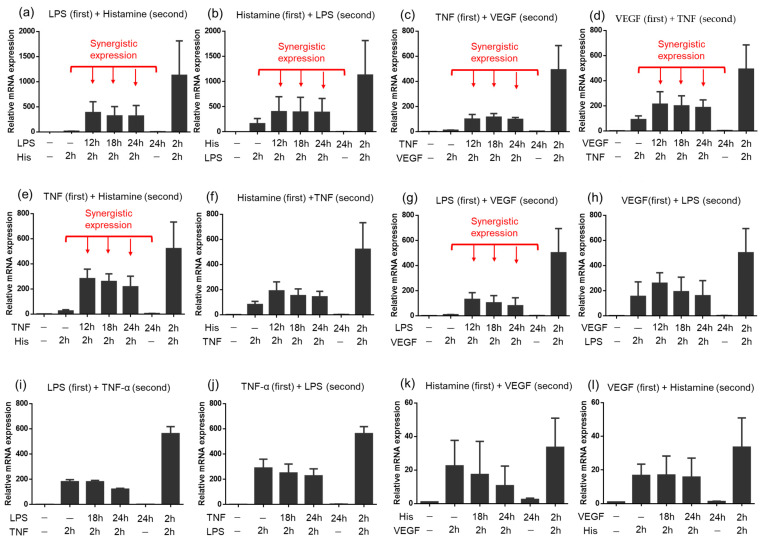
Priming effects of TFIs on TF mRNA expression in HUVECs. HUVECs were treated with a TFI (first stimulation) and then stimulated with other TFIs (second stimulation) for duration indicated in each panel at indicated time as shown in Figure S1. Concentrations of each molecule are VEGF (10 ng/mL), TNF-α (10 ng/mL), LPS (100 ng/mL), and/or histamine (10 μM). (**a**) LPS (first) + Histamine (second), (**b**) Histamine (first) + LPS (second), (**c**) TNF–α (first) + VEGF (second), (**d**) VEGF (first) + TNF-α (second), (**e**) TNF-α (first) + Histamine (second), (**f**) Histamine (first) + TNF-α (second) (**g**) LPS (first) + VEGF (second), (**h**) VEGF (first) + LPS (second), (**i**) LPS (first) + TNF-α (second), (**j**) TNF-α (first) + LPS (second), (**k**) Histamine (first) + VEGF (second), (**l**) VEGF (first) + Histamine (second). Time course of TF expression by each stimulation is shown in Figure 1. Red texts and arrows show synergistic effect of co-stimulation by two TFIs (S/A > 2). All figures show results of three independent experiments. Data represent mean ± standard error.

Considering that vascular endothelial cells under physiological condition are not likely to be stimulated with both groups of TFIs at the same time, we then investigated the priming effect of each TFI for the expression of TF in HUVECs. We first stimulated HUVECs with a TFI (first stimulation) and then stimulated the cells with another TFI at indicated time points (second stimulation). Time courses of cell stimulation are summarized in Figure S1. We here evaluated if the TF expression was synergistic or additive using the formula described in the Section 4 [21]. The TF mRNA expression levels and calculation of S/A ratios are calculated and summarized in Figure S2a,b. The synergistic TF mRNA expression was observed even when the second stimulation (different group) was added at 10, 16 or 22 h after the first stimulation (Figure 3a–h and Figure S2a). The level of TF expression in response to histamine (first stimulation) followed by TNF-α (second stimulation) and that to VEGF (first stimulation) followed by LPS (second stimulation) (Figure 3f,h and Figure S2a,b) were relatively weak and did not reach the definition of synergistic expression. However, TF was expressed beyond the level of an additive effect when HUVEC was stimulated by the same combination of inducers but in reverse order, i.e., LPS in the first stimulation and VEGF in the second one. Although the other combinations of a stimulant in Group 1 and another in Group 2 TFIs showed synergistic expressions of TF, a similar tendency was observed in the orders of addition of stimulants, suggesting that the priming effect by TFIs in Group 1 is potent compared with that in Group 2. Moreover, priming effects were not observed when HUVECs were stimulated with TFIs of the same group, such as combinations with LPS and TNF-α, or with histamine and VEGF (Figure 3i–l and Figure S3). Thus, the priming effect of Group 1 TIs on HUVECs lasts for longer than a half day and synergistically enhanced TF expression triggered by stimuli by Group 2 TFIs.

**Figure 4:**
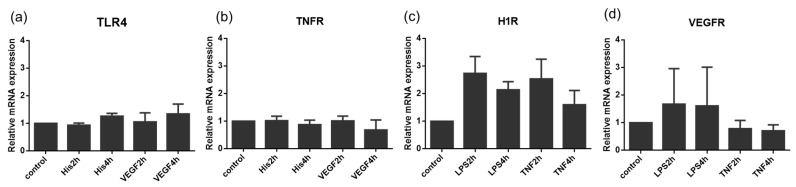
Expression levels of mRNA of receptors in HUVECs in response to TFIs. Effects of TFIs on the mRNA expression of (**a**) TLR4, (**b**) TNFR, (**c**) H1R, and (**d**) VEGFR in HUVECs. All figures show results of three independent experiments. Data represent mean ± standard error.

To explore the mechanism of priming effects of the treatment with TFIs, we then studied the effects of TFI on the upregulation of each receptor. As shown in Figure 4, treatment with LPS or TNFα increased the expression of mRNA for H1R but not for VEGFR in HUVECs. Treatment with histamine or VEGF did not increase mRNA expression of TLR-4 or TNFR (Figure 4). Moreover, treatment with LPS or TNF did not increase the expression of mRNA for VEGFR. Therefore, the upregulation of H1R may be involved, but not likely crucial, in the priming effects of TF expression by vascular endothelial cells.

### 2.2. Time-Dependent Expression of TF on the Surface of HUVECs and Its Activity as a Trigger of the Extrinsic Coagulation Pathway and Gap Formation of HUVECs

**Figure 5:**
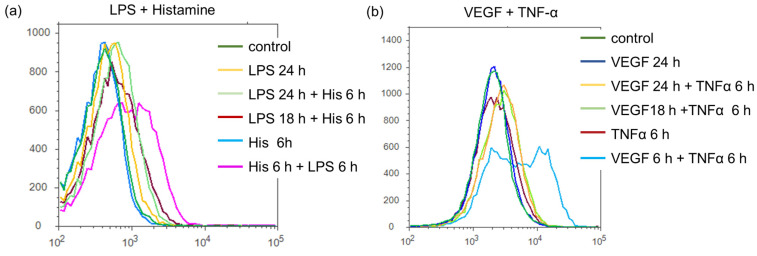
Expression levels of TF on the surface of HUVECs. TF expression on the surface of HUVECs in response to indicated combination of TFIs. VEGF (10 ng/mL), TNF-α (10 ng/mL), LPS (100 ng/mL), and/or histamine (10 μM) were stimulated for duration indicated in a subfigure of each panel at indicated time points as shown in Figure S4. All figures show a representative result of three independent experiments.

We then investigated the priming effect of TFIs on TF expression on the surface of plasma membrane of HUVECs by using flow cytometry. Timings of each stimulation are summarized in Figure S4. Since peak TF expression on plasma membrane was around 6 h after stimulation according to our previous study, we here detected TF expression levels 6 h after the second stimulation. As shown in Figure 5, the priming effects of TFIs were observed when HUVECs were stimulated with TFI (second stimulation) even after 12 or 18 h of the first stimulation (a: light green and red lines, b: yellow and light green lines).

**Figure 6:**
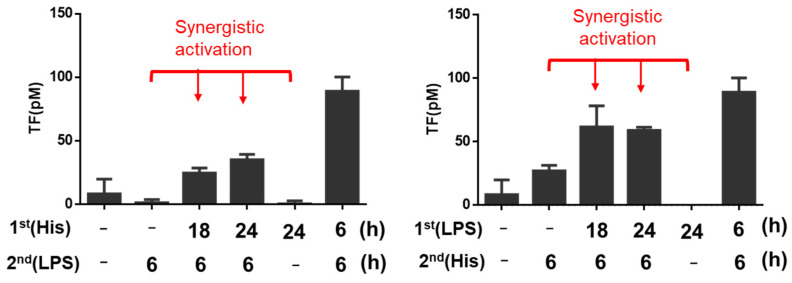
Priming effect of TFIs on the TF activity expressed on the surface of HUVECs. The activity of TF expressed on cells increased when HUVECs were stimulated with different group of TFIs, LPS (100 ng/mL), and histamine (10 μM) at indicated time points, as shown in Figure S4. Red texts and arrows show synergistic effect of co-stimulation by two TFIs (S/A > 2). The figure shows a representative result of three independent experiments. Data represent mean ± standard error.

We then investigated the priming effect of TFIs on TF-triggered activation of the coagulation pathway by using TF actichrome^®^ TF activity kit. Each TF activity was also measured 6 h after the second stimulation. As shown in Figure 6, TF activity expressed on the surface of HUVECs was also synergistically increased even when the second stimulation with different groups of TFIs was added 12 or 18 h after the first stimulation of TFI (Figure 6).

**Figure 7:**
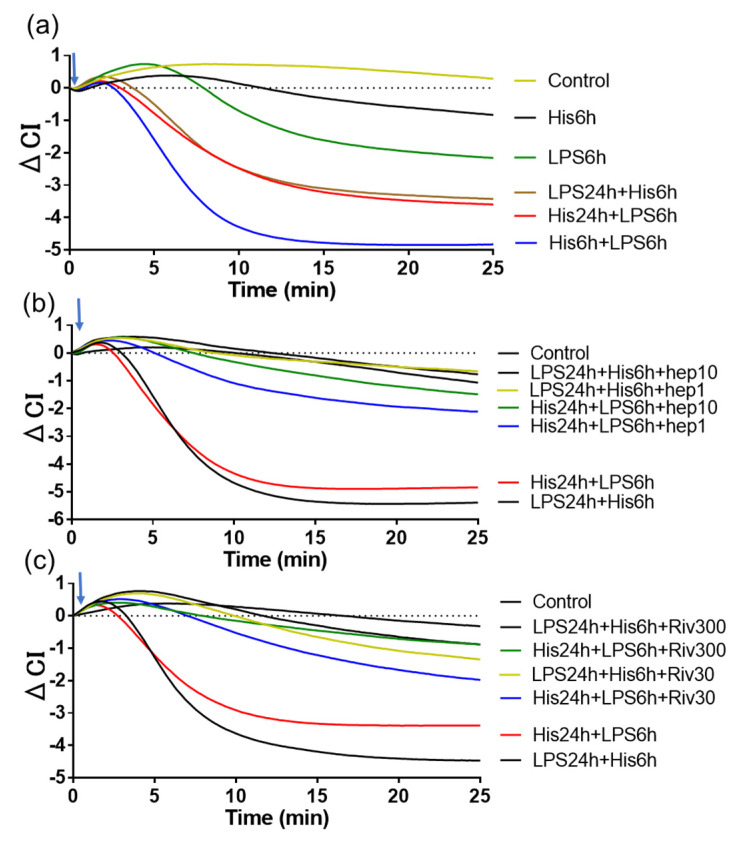
Priming effect of TFIs and anticoagulants on the gap formation of HUVECs. Real time evaluation of gap formation of TF expressing HUVECs in the presence of VIII-deficient plasma. (**a**) Measurement of gap formation of HUVECs which express TF in response to histamine (10 μM) and LPS (1 ng/mL) at indicated time as shown in Figure 6. Effects of anticoagulant drugs (**b**) heparin (1, 10 U/mL) and (**c**) rivaroxaban (30, 300 ng/mL) on the TF-induced gap formation of HUVECs. Each figure shows a representative result of three independent experiments.

Finally, we investigated if synergistic expression of TF expressed on HUVECs in the presence of plasma including extrinsic coagulation factors, including factor VII, X, and II but not factor VIII, induced gap formation of HUVECs by using an impedance sensor. In this method, a decrease in CI shows the gap formation of HUVECs. Gap formation was measured 24 h after the first stimulation according to our previous experiments. As shown in Figure 7a, intra-cellular gap formation of HUVECs (CI decrease) was found when the second stimulation of TFI was added to HUVECs as late as 18 h after the first stimulation. Moreover, the synergistic expression of TF-induced gap formation of HUVECs was clearly suppressed by the treatment with anticoagulation molecules heparin and rivaroxaban (Xa inhibitor) (Figure 7b,c).

## 3. Discussion

In this study, we demonstrated that synergistic TF expression (mRNA and surface protein), TF activity as a trigger of the extrinsic coagulation cascade, and gap formation of vascular endothelial cells were synergistically increased even when the second stimulation was added as late as 18 h after the first stimulation, respectively. Moreover, gap formation of HUVECs stimulated by TFIs with a time lag were effectively blocked by the treatment with anticoagulant drugs heparin and rivaroxaban, suggesting that the reactions are also induced by the TF-triggered extrinsic coagulation pathway. Until now, several molecules in blood have been considered as possible biomarkers to reflect the severity and activity of CSU. These biomarkers include VEGF, histamine, D-dimer, FDP, (PF_1+2_), C-reactive protein (CRP), substance P (SP), and also proinflammatory cytokines such as TNF-α and interleukins such as IL-1β, -4, -6, -17, -31 and -33, in peripheral blood of patients with CSU [22,23,24,25,26,27]. Moreover, infections by bacteria and/or virus have been suggested to be associated with the pathogenesis of CSU. It is widely accepted that wheal formation of urticaria is due to skin mast cell activation, but it has not been clarified how these molecules induce intermittent rather than continuous activation of mast cells in the skin/basophils in peripheral blood and wheal formation in the clinical setting. We have demonstrated that histamine and several cytokines or certain toll-like receptor agonists, such as LPS, may prime vascular endothelial cells. The endothelial cells have a maintained susceptibility for a half day or longer to the second stimulation by a factor in the other group for TF expression to activate the vascular coagulation system, which then activates peripheral basophils and skin mast cells specifically in the skin through formation of complement C5a, produced by activated coagulation factors, rather than by classical or alternative complement pathways. We further revealed that the first stimuli on HUVECs by LPS or TNF stimulation enhanced the expression of mRNA for H_1_R, implying that TFIs in Group 1 may affect both the numbers and/or functions of other membrane-associated and/or intra-cellular molecules of vascular endothelial cells. The time course of wheal formation in CSU is slow and lasts for hours, whereas that induced by bullous injection of histamine disappears within an hour or so [28]. These observations suggest the involvement of an additional mechanism to induce mast cell activation for a certain duration, most likely for hours, in the skin. The redundancy and duration of the first and the second stimuli, which affect various molecules with different susceptibilities to medications, such as antihistamine, immunosuppressants, and corticosteroids, are in line with a large variability in clinical efficacy of medications among CSU patients.

Recently, we have demonstrated that the concentrations of IgE antibody and histamine in peripheral blood of CSU patients are significantly higher than that in serum of healthy donors [29,30]. High concentrations of IgE (>1 μM) activate IgE-depleted human peripheral basophils and induce histamine release in the absence of any antigens (allergens) [29]. Moreover, we reported that spontaneous release of histamine from basophils of CSU patients is higher than that of healthy donors, suggesting that basophils themselves may contribute to the synergistic expression of TF on vascular endothelial cells and the activation of the extrinsic coagulation cascade [30]. TF-induced activation of coagulation pathway produces active forms of coagulation/fibrinolysis factors (factor Xa, factor IIa and plasmin), which then convert complement 5 (C5) to C5a, anaphylatoxin, by their protease activity, an active secretagogue for peripheral basophils and skin mast cells via C5a receptor (C5aR) [4,31]. Thus, the exposure of vascular endothelial cells to various combinations of TFIs within a certain duration may induce intercellular gap formation first by direct action of the activated coagulation factors followed by a leakage of plasma containing C5a which is produced by a positive loop of the coagulation and complement system and selectively activates skin mast cells and basophils, resulting in a long-lasting and robust edema formation, recognized as uticarias in patients with CSU.

## 4. Materials and Methods

### 4.1. Reagents

LPS, endothelial cell growth supplement (ECGS), heparin, and bovine serum albumin (BSA) were purchased from Sigma-Aldrich Japan (Tokyo, Japan). Anti-TF-FITC and Actichrome^®^ TF activity assay kit was purchased from SEKISUI DIAGNOSTICS (Lexington, MA, USA). HUVECs from ATCC (Manassas, VA, USA). Coagulation factor VIII deficient human plasma was purchased from COSMO BIO Co., Ltd. (Tokyo, Japan). DMEM/F12 was purchased from Thermo Fisher Scientific, Inc. (Waltham, MA, USA). Histamine was from Wako Pure Chemical Industries, Ltd. (Osaka, Japan). Recombinant human TNF-α and VEGF were purchased from R & D systems (Minneapolis, MN, USA). RepCell^®^ was purchased from CellSeed Inc. (Tokyo, Japan)

### 4.2. Cells

HUVECs were cultured in DMEM/F12 supplemented with 10% fetal calf serum (FCS), 100 U/mL penicillin, 100 μg/mL streptomycin, 40 μg/mL ECGS, and heparin (0.1 mg/mL). The day before experiments, HUVECs were harvested using trypsin and cultured in 96-well plates for TF activity assay, 24-well plates for mRNA isolation, for flow cytometry, and E-plate for impedance assay.

### 4.3. Quantitative PCR

Total RNA was isolated from HUVECs according to instruction of the RNeasy Mini kit (Qiagen, Venlo, The Netherlands), and cDNA was generated according to the instruction of the QuantiTect Reverse transcription kit (Qiagen). Levels of mRNA expression of TF and GAPDH (internal control) were analyzed using quantitative PCR (qPCR) using ABI 7300 Real time PCR system (Applied Biosystems, Carlsbad, CA, USA) with PCR reagent, Power SYBR Green PCR Master Mix (Applied Biosystems). The specific primer pairs were as follows: forward primer for TF, 5′-GGAACCCAAACCCGTCAATC-3′; reverse primer for TF, 5′- GTCCGAGGTTTGTCTCCAGGTA-3′, and forward primer for GAPDH, 5′-GAAGGTGAAGGTCGGAGTCA-3′; reverse primer for GAPDH, 5′-GAAGATGGTGATGGGATTTCC-3′. The expression of GAPDH was measured as an internal control for calibration of expression level of target gene. Levels of mRNA expression of H1R, VEGFR, TNFR TLR4, and GAPDH were also evaluated by TaqMan^TM^ Gene Expression Assay using following reagents, 01052961_m1 for VEGFR1, Hs00152939_m1 for TLR4, Hs01042313_m1 for TNFR, Hs 00269693_s1 for H1R, and Hs02758991_g1 for GAPDH) by means of ABI 7300 Real time PCR system.

#### Evaluation of Synergistic or Additive Expression of TF mRNA

Synergistic expression/additive expression (S/A) ratio, which we defined previously, was used for the evaluation of synergistic or additive expression effect of the combination of TFIs on mRNA expression of TF in HUVECs. The S/A ratio, which we defined previously, was calculated according to the following mathematical formula [21].

S/A ratio = [mRNA expression of TF by A+B]/

[mRNA expression of TF by A] + [mRNA expression of TF by B]

When the combination of TFIs shows only additive effect for TF expression, the S/A ratio is around 1. On the other hand, when co-stimulation with TFIs shows a synergistic effect for TF expression, the S/A ratio is substantially higher than 1. We here defined synergistic effect as 2 or more of S/A ratio.

### 4.4. Analysis Using Flow Cytometry

Expression levels of TF on the surface of HUVECs after 6 h of the second stimulation were measured by flow cytometric analysis as described in a previous manuscript [21]. Timings of each stimulation are summarized in Figure S4. HUVECs cultured in RepCell^®^ were stimulated and then collected by temperature reduction. Collected cells were treated with FITC labeled anti-human TF (American Diagnostica, Stamford, CT, USA) or FITC-conjugated mouse IgG1 without any labeling (Myltenyi Biotec, North Rhine-Westphalia, Germany) as an isotype control. The f luorescence intensity of individual cells was analyzed using an Attune acoustic focusing cytometer (Life technologies, Tokyo, Japan).

### 4.5. TF Activity Assay

The activity of TF expressed on the surface of cells was measured according to the instructions of an Actichrome^®^ TF activity assay kit, as described in our previous work [21]. Briefly, HUVECs were cultured in a culture plate over-night. Timings of stimulations are shown in Figure S4. The cells were washed and then exposed to an assay buffer containing coagulation factor VIIa and coagulation factor X. After incubation, spectrozyme factor Xa substrate was added and incubated for 30 min at 37 °C. Absorbance at 405 nm of each well was measured using a plate reader VARIOSKAN Flash (Thermo Fisher Scientific, Inc.).

### 4.6. Impedance-Based Analysis of Vascular Permeability (Gap Formation of HUVECs)

Impedance analysis was performed as described before [21]. Briefly, HUVECs were seeded in E-Plates (Roche Applied Science, Upper Bavaria, Germany) at a density of 50,000 cells per well. Then cells were treated with LPS, TNF-α, VEGF, and/or histamine at the indicated time points. Timing of stimulations are shown in Figure S5. On Day 3, cells in each well were washed and treated with culture medium with 2% BSA without any supplements. The E-plate was then set onto iCElligence (Roche Applied Science), and cell index (CI) was measured every 10 s in the presence of 1% factor VIII-deficient human plasma.

## 5. Conclusions

We clarified that combinations of different groups of TFIs synergistically express TF on the surface of vascular endothelial cells even if a time lag exceeds 18 h, resulting in the activation of the extrinsic blood coagulation cascade, producing active forms of coagulation factors, and finally inducing gap formation between vascular endothelial cells (plasma leakage). Further studies on the priming effects of TFIs are warranted to clarify the detailed pathogenesis of CSU and assist in the development of effective drugs for refractory CSU.

## Data Availability

Data presented by this article are available from the corresponding author up on reasonable requests.

## Supplementary Materials

The following supporting information can be downloaded at: https://www.mdpi.com/article/10.3390/ijms241512388/s1.
