# Supplementary material for: Time Course of Priming Effect of TF Inducers on Synergistic TF Expression and Intra-Cellular Gap Formation of Human Vascular Endothelial Cells via the Extrinsic Coagulation Cascade
Source: Int J Mol Sci. 2023 Aug 3;24(15):12388. doi: 10.3390/ijms241512388 (PMC10419186; doi:10.3390/ijms241512388)
Supplement: Supplementary file 1 [file ijms-24-12388-s001.zip › ijms-2501931-supplementary.pdf]

Figure S1: Time courses of stimulation of HUVECs with TFIs for TF mRNA expression assay.

Figure S2: Summarized results of (a) TF mRNA expression levels and (b) calculated S/A ratios in response to TFIs (first stimulation) and/or second stimulation.

Figure S3 Summarized results of (a) TF mRNA expression levels and (b) calculated S/A ratios in HUVECs in response to the same group of TFIs.

Figure S4: Time courses of stimulation of HUVECs with TFIs for TF activity assay and flow cytometry.

Figure S5: Time courses of stimulation of HUVECs with TFIs for impedance assay. Figure S5a is for figure 6 (a), and figure S5b is for figure 6 (b)(c).

Figure S1

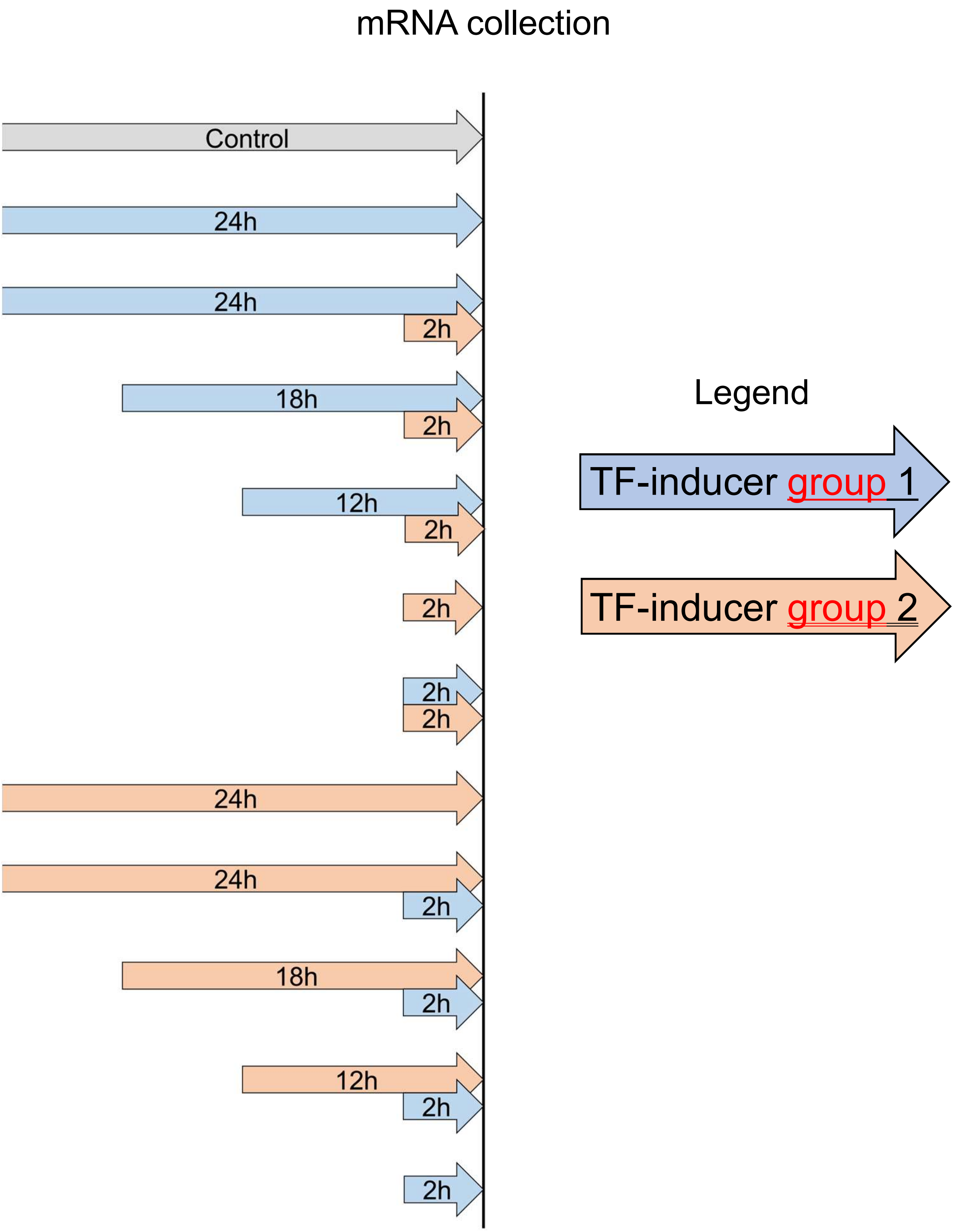
mRNA collection
Control
24h
24h
2h
18h
2h
12h
2h
2h
2h
2h
24h
24h
2h
18h
2h
12h
2h
2h
Legend
TF-inducer group 1
TF-inducer group 2

Figure S2a

	No stimulus	His 2h	His 24h
No stimulus	1	12.1	1.8
LPS 2h	160.9	1126.2	385.9
LPS 24h	1.9	315.5	

	No stimulus	VEGF 2h	VEGF 24h
No stimulus	1	10.7	3.1
TNF-α 2h	90.5	491.4	186.4
TNF-α 24h	3.4	97.7	

	No stimulus	LPS 2h	LPS 24h
No stimulus	1	154.3	1.4
VEGF 2h	8.3	500.7	79.7
VEGF 24h	1.7	159.4	

	No stimulus	TNF-α 2h	TNF-α 24h
No stimulus	1	81.0	3.6
His 2h	24.2	521.2	217.3
His 24h	1.7	142.1	

Figure S2b

S/A ratio= [TF mRNA expression by A+B] /
[TF mRNA expression by A] + [TF mRNA expression by B]

S/A ratio= [stimulus LPS 24h + His 2h] /
[single stimulus LPS24 h]+[single stimulus His2 h]
=315.5/1.9+12.1 =22.5

S/A ratio= [stimulus His 24h + LPS 2h] /
[single stimulus His24 h]+[single stimulus LPS 2h]
=385.9/1.8+160.9 =2.37

S/A ratio= [stimulus TNF-α 24h + VEGF 2h] /
[single stimulus TNF-α 24 h]+[single stimulus VEGF 2h]
=97.7/3.4+10.7 =6.93

S/A ratio= [stimulus VEGF 24h + TNF-α 2h] /
[single stimulus VEGF 24 h]+[single stimulus TNF-α 2h]
=188.4/3.1+90.5 =2.01

S/A ratio= [stimulus LPS 24 h + VEGF 2h] /
[single stimulus LPS 24 h]+[single stimulus VEGF 2h]
=79.7/1.4+8.3 =8.22

S/A ratio= [stimulus VEGF 24h + LPS 2h] /
[single stimulus VEGF 24 h]+[single stimulus LPS 2 h]
=159.4/1.7+154.3 =1.02

S/A ratio= [stimulus TNF-α 24 h + His 2 h] /
[single stimulus TNF-α 24 h]+[single stimulus His 2h]
=217.3/3.6+24.2=7.82

S/A ratio= [stimulus His 24 h + TNF-α 2h] /
[single stimulus His 24 h]+[single stimulus TNF-α 2h]
=142.1/1.7+81.0=1.72

Figure S3

	No stimulus	LPS 2h	LPS 24h
No stimulus	1	289.2	2.9
TNF-α 2h	180.2	561.4	120.8
TNF-α 24h	2.9	227.1	

	No stimulus	VEGF 2h	VEGF 24h
No stimulus	1	22.5	1.3
His 2h	16.7	33.5	15.7
His 24h	2.5	10.7	

S/A ratio= [stimulus LPS 24h + TNF-α 2h] /
[single stimulus LPS 24h]+[single stimulus TNF-α 2h]
=120.8/2.9+190.2 =0.63

S/A ratio= [stimulus TNF-α 24h + LPS 2h] /
[single stimulus TNF-α 24h]+[single stimulus LPS 2h]
=227.1/2.9+289.2 =0.78

S/A ratio= [stimulus VEGF 24h + His 2h] /
[single stimulus VEGF 24h]+[single stimulus His 2h]
=15.7/1.3+16.7=0.87

S/A ratio= [stimulus His 24h + VEGF 2h] /
[single stimulus His 24h]+[single stimulus VEGF 2h]
=10.7/2.5+22.5=0.43

Figure S4

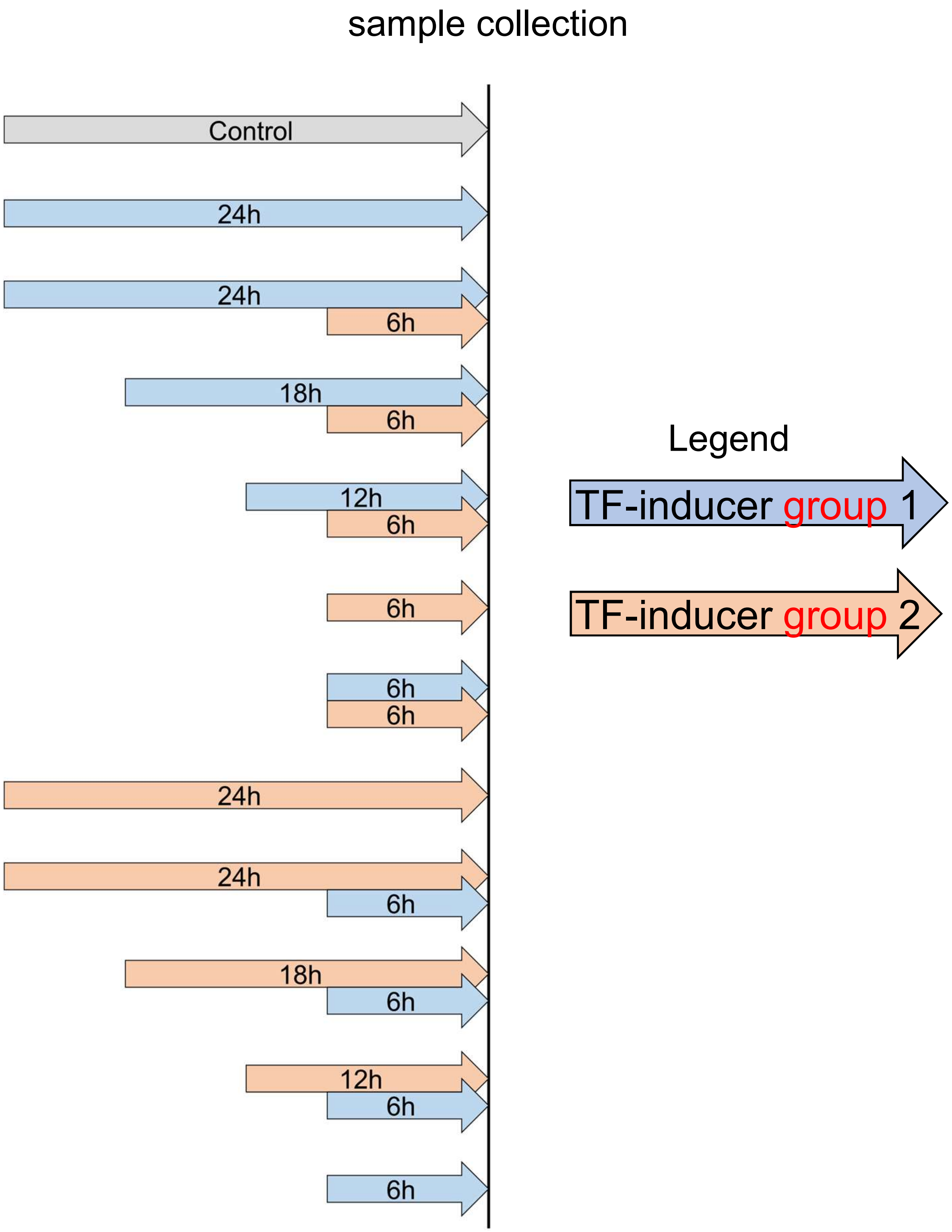
sample collection
Control
24h
24h
6h
18h
6h
12h
6h
6h
6h
6h
24h
24h
6h
18h
6h
12h
6h
6h
Legend
TF-inducer group 1
TF-inducer group 2

Figure S5a

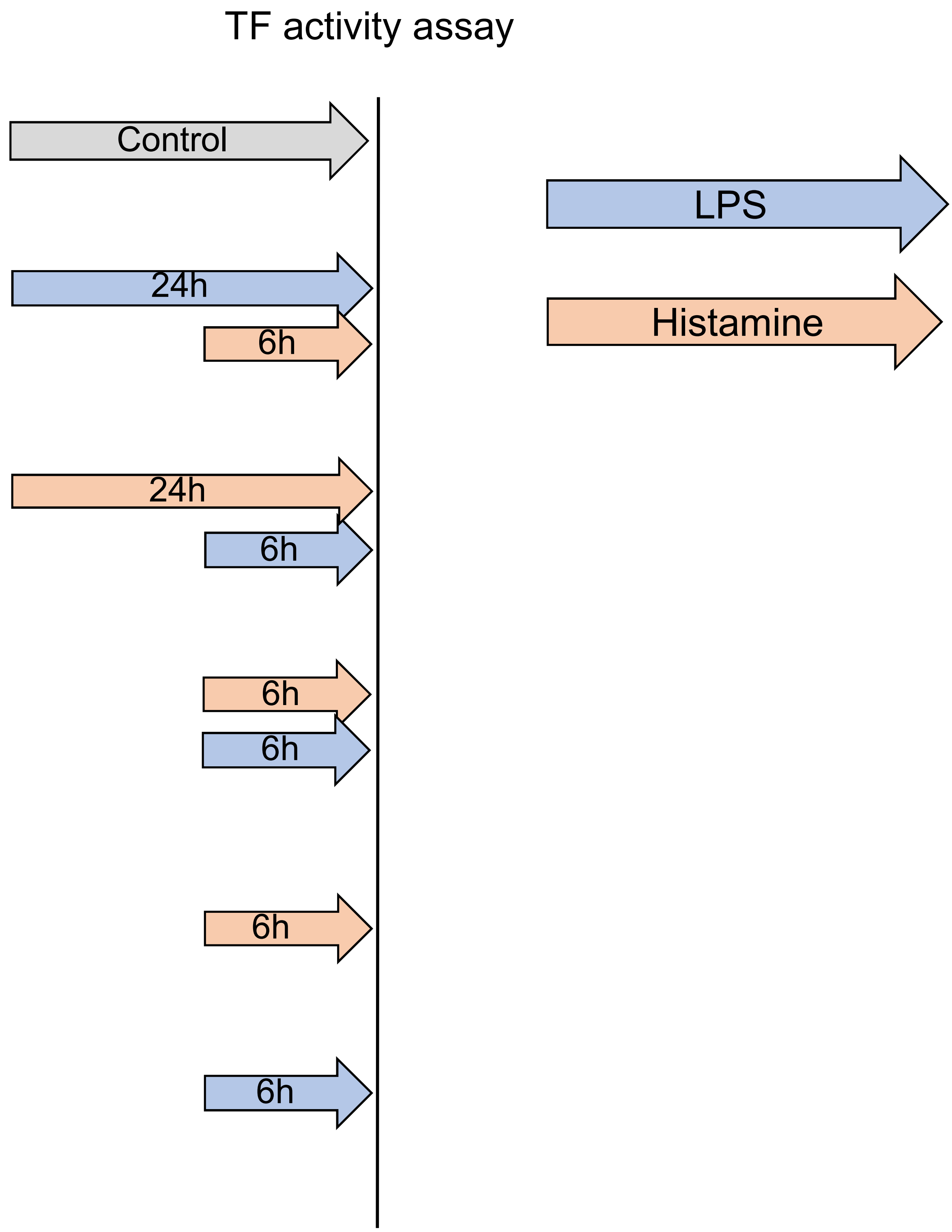
TF activity assay
Control
LPS
24h
6h
Histamine
24h
6h
6h
6h
6h
6h

Figure S5b

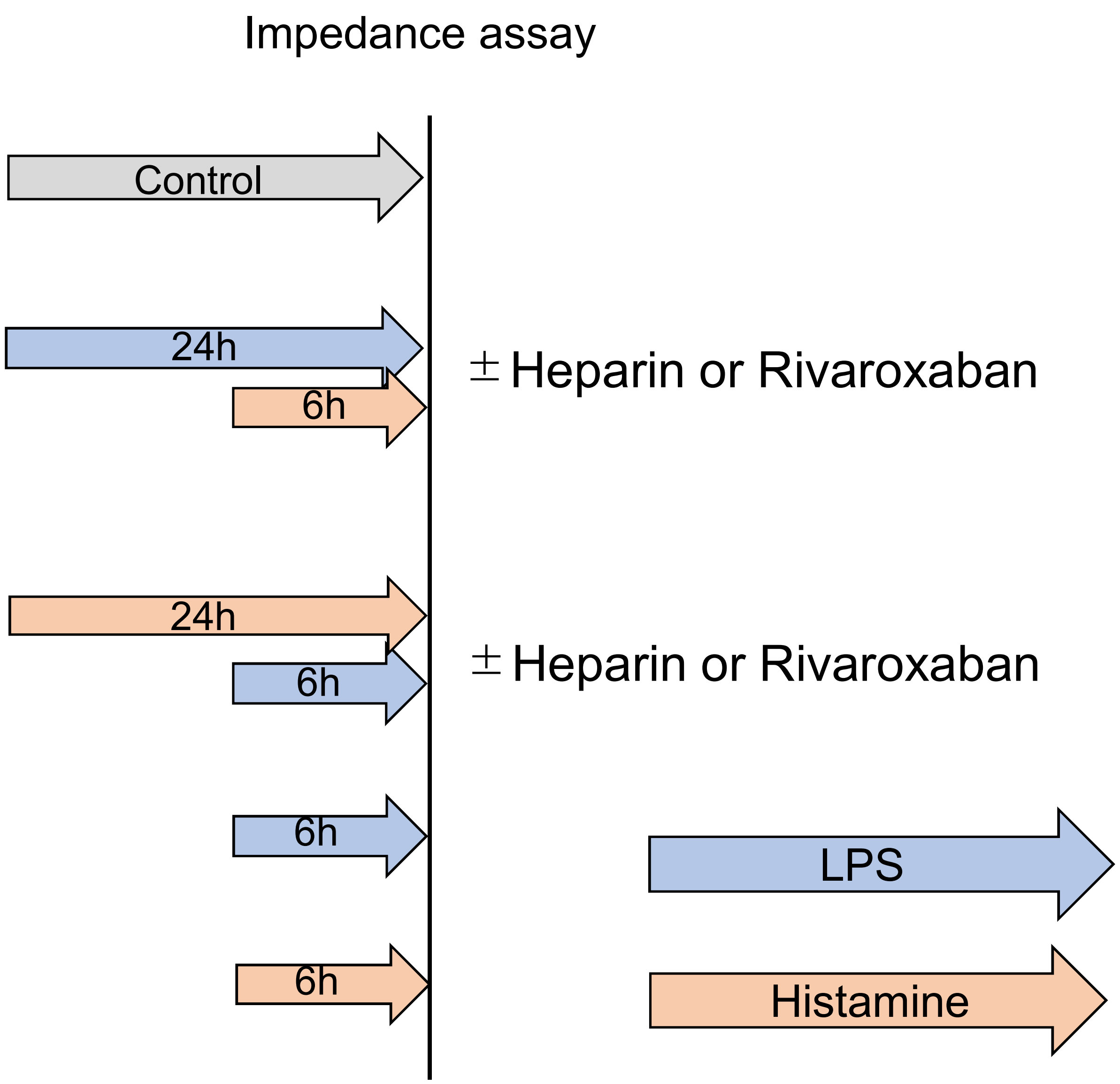

Impedance assay
Control
24h
6h
± Heparin or Rivaroxaban
24h
6h
± Heparin or Rivaroxaban
6h
6h
LPS
Histamine
